# Complete mitochondrial genome of the Grey-capped Greenfinch subspecies, *Chloris sinica ussuriensis* (Passeriformes: Fringillidae)

**DOI:** 10.1080/23802359.2018.1467234

**Published:** 2018-06-22

**Authors:** Joo-Eun Kim, Jong-Gil Park, Kyoung-Soon Jin, Chungoo Park, Dong-Ha Nam

**Affiliations:** a Department of Biological Sciences, College of Natural Sciences, Chonnam National University, Gwangju, Korea;; b Birds Research Center, Korea National Park Research Institute, Korea National Park Service, Jeollanam-do, Korea;; c School of Biological Sciences and Technology, College of Natural Sciences, Chonnam National University, Gwangju, Korea

**Keywords:** Chloris sinica ussuriensis, Fringillidae, mitochondrial genome

## Abstract

We sequenced the complete mitochondrial (mt) genome of *Chloris sinica ussuriensis*. The circular mt genome is 16,813 bp long and encodes 13 proteins, 22 transfer RNAs, and 2 ribosomal RNAs. Phylogenetic analysis based on full mt genome sequences confirmed that the *C. s. ussuriensis* is monophyletic group of the *Chloris sinica*. The complete mitochondrial genome of *C. s. ussuriensis* can provide a valuable data for resolving geographical distribution of evolutionary subdivision within the *C. sinica* species in East Asia.

The Grey-capped Greenfinch *Chloris sinica* (Aves: Passeriformes: Fringillidae) is a small passerine bird in the Fringillidae family, which has limited ranges of distribution in East Asia. This species is now regarded as consisting of five to six subspecies (del Hoyo et al. 2010; Gill and Donsker 2014), but the phylogenetic relationships are still unclear and additional taxon sampling and sequence data are needed. Here, we present the complete mitochondrial (mt) genome of *C.* s. *ussuriens*is to help elucidate the phylogeographic patterns of the *C. sini*ca species.

During the breeding season, the *C. s. ussuriensis* specimen was collected from Taean, Chungcheong-do, Korea. We extracted the genomic DNA from the blood sample using the DNeasy Blood & Tissue kit (Qiagen, Valencia, CA) as described by the manufacturer’s protocol, and determined the complete mt genome sequence using the primer-walking approach. The blood sample used in this study was deposited at the Wildlife Specimen Bank in Chonnam National University, Korea.

The complete mt genome of *C. s. ussuriensis* was 16,813 bp in length (GenBank accession No. MH047559), and consisted of 13 protein-coding genes, 22 transfer RNA (tRNA) genes, 2 ribosomal RNA genes (rRNAs), an origin of light strand replication site (O_L_), and a putative long noncoding region called the control (D-loop) region, agreeing with the typical vertebrate gene arrangement. Most of the mt genes were encoded on the H-strand, with the exception of the one protein-coding gene (*nad6*) and eight tRNA genes (*tRNA^Gln^*, *tRNA^Ala^*, *tRNA^Asn^*, *tRNA^Cys^*, *tRNA^Tyr^*, *tRNA^Ser^*, *tRNA^Pro^*, and *tRNA^Glu^*) that were encoded on the L-strand. The overall nucleotide composition for *C. s. ussuriensis* was 30.7% A, 30.5% C, 14.1% G, and 24.7% T.

The phylogenetic analysis of full mt genome of *C. s. ussuriensis* with two available complete mt genomes from *Chloris* genus and nine ecologically close species revealed that *C. s. ussuriensis* was the most closely related to the *Chloris sinica* (Figure 1). The present study provides essential mt genome data for further intraspecific phylogeography and population differentiation among the Grey-capped Greenfinches in East Asia.

**Figure 1.**
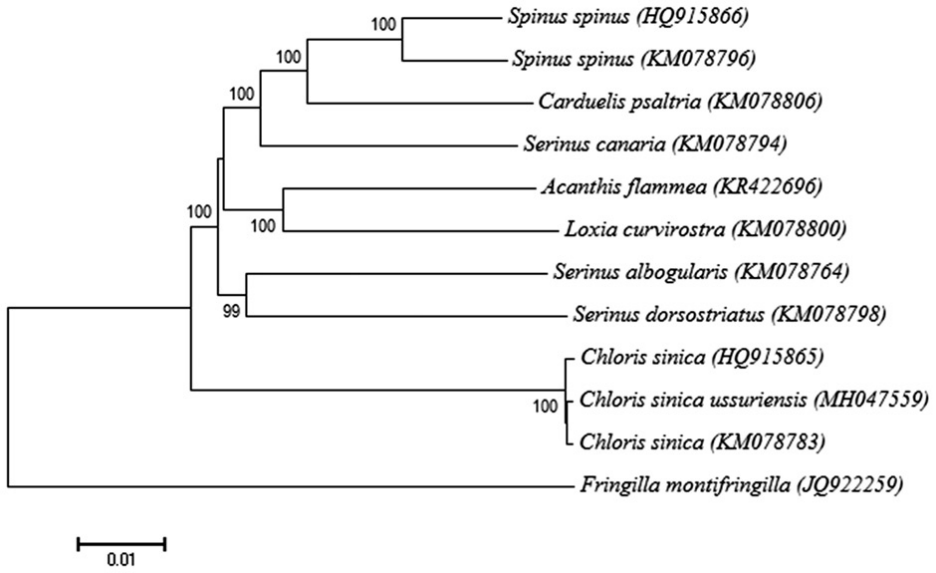
Phylogeny of *Chloris sinica ussuriensis* and other related species with *Fringilla montifringilla* as outgroup. The phylogenetic tree derived from complete mitochondrial (mt) genome sequences was constructed by a neighbour-joining method with 1000 bootstrap replicates in the program MEGA7 (Saito and Nei 1987). GenBank accession numbers of each mt genome sequence are given in the bracket after the species name. In accordance with the recently proposed nomenclature (Zuccon et al. 2012), in this study, *Carduelis sinica* was named as *Chloris sinica*.

GenBank accession number from the complete mitochondrial genome of Chloris sinica ussuriensis (MH047559) has been registered with the NCBI database.
